# An Experimental Study on Flow and Heat Transfer Characteristics of Ethanol/Polyalphaolefin Nanoemulsion Flowing Through Circular Minichannels

**DOI:** 10.1186/s11671-017-1984-1

**Published:** 2017-03-23

**Authors:** Vu Trinh, Jiajun Xu

**Affiliations:** Department of Mechanical Engineering, University of the District of Columbia, Washington, DC 20008 USA

**Keywords:** Nanoemulsion, Convective heat transfer, Phase change, Minichannel

## Abstract

This work experimentally studied the convective flow and heat transfer characteristics of a novel nanostructured heat transfer fluid: “ethanol/polyalphaolefin nanoemulsion” flowing through 12 circular minichannels of 1-mm diameter each. Ethanol/polyalphaolefin nanoemulsion is a thermodynamically stable system formed by dispersing ethanol into a mixture of “polyalphaolefin (PAO)” and surfactants. In this study, ethanol/PAO nanoemulsion is used as the working fluid to study the effect of ethanol nanodroplets on its convective flow and heat transfer characteristics. In addition, the effect of flow regime on its heat transfer is examined. It is found that using ethanol/PAO nanoemulsion fluids can improve convective heat transfer compared to that of pure PAO under both single- and two-phase flow regimes. For single-phase flow, there is no significant difference in Nusselt number between ethanol/PAO nanoemulsion and pure PAO in laminar flow regime. However, when entering transition flow regime, the ethanol/PAO nanoemulsion fluid showed a substantial increase in Nusselt number. Meanwhile, there is an increase in pressure drop and early onset of the laminar-turbulent transitional region for the ethanol/PAO nanoemulsion compared to pure PAO. The heat transfer coefficient of ethanol/PAO nanoemulsion can be further enhanced when the ethanol nanodroplets undergo phase change, which is hypothesized that such an effect is likely related to the enhanced interfacial thermal transport between the nanodroplets and base fluid under elevated temperature and the latent heat of phase changeable nanodroplets inside nanoemulsion. Further studies are needed to fully explore the convective heat transfer properties of nanoemulsion fluids.

## Background

The thermal management challenges faced in various industries have urged the heat transfer community to develop novel thermal management solutions including more efficient heat exchangers and new heat transfer fluids with significantly improved thermal properties [1–5]. To date, although more advanced works have been performed to develop high performance heat exchangers with varieties of shape, size, and tube surface augmentation, heat transfer fluids with significantly improved thermal properties over those currently available remain one of the most challenging tasks. Nevertheless, there are several heat transfer fluid candidates reported such as nanofluid [6–29], dilute emulsion [30, 31], and emulsion [32–39]: Nanofluid was proposed in 1995 by Choi [40], and since then, it has been intensively studied for its preparation, phase stability, and its potential applications in high heat flux cooling such as nuclear power system, solar collector, and compact high power density electronics system. An emulsion fluid is a mixture of two immiscible liquids in which one liquid (the dispersed or droplet component) forms a suspension of many small droplets in the other liquid phase (the continuous component). Using emulsion to enhance heat transfer can be dated back to 1959 by Moore [41]. Later on, the emulsion of 5 vol% or less dispersed component is called “dilute emulsion,” and it has attracted interests from several research groups [30–36, 42, 43]. However, the understanding of phase change heat transfer inside emulsion is limited. One of the most detailed descriptions of how emulsions boil is the work of Bulanov and Gasanov [33–36, 42, 43], in which they proposed chain-reaction boiling of the droplets as an explanation for the observed superheated droplets and bubble dynamics on the heat surface. To further understand the boiling mechanism of dilute emulsions, Rosele et al. [44] carried out an experimental study of boiling heat transfer from a horizontal heated wire, including visual observations in which the heat transfer coefficient is enhanced in dilute emulsions compared to that of water as a base fluid. Aside from all the efforts mentioned above, recently, the author has proposed a radically new design for thermal fluids called “nanoemulsion.” The nanoemulsion fluids completely eliminate solid particles, which usually cause abrasion and erosion problem issues even with extremely fine particles such as nanoparticles [45–49], and instead use liquid nanostructures [50–59]. Nanoemulsion is a suspension of liquid nanodroplets in fluids, which is part of a broad class of multiphase colloidal dispersions [60]. The droplets typically have a length scale less or equal to 50 nm, which makes the nanoemulsion transparent to natural light. With the addition of another liquid into base fluid to form droplets of nanometers, the overall heat transfer characteristics of the system are expected to be improved especially with the phase change of nanodroplets. A comparison of nanoemulsion with emulsion (dilute emulsion) is shown in Table 1 [60].

**Table 1:** Comparison of nanoemulsion and emulsion (dilute emulsion)

Property	Nanoemulsion	Emulsion (dilute emulsion)
Appearance	Transparent	Turbid
Interfacial tension	Ultra-low (usually ≪1 mN/m)	Low
Droplet size	<50 nm	>500 nm
Phase stability	Thermodynamically stable, long shelf life	Thermodynamically unstable
Preparation	Self-assembly	Need of external shear
Viscosity	Newtonian	Non-Newtonian

Previous studies of nanoemulsion have shown that the thermophysical properties of the nanoemulsion are better than that of the base fluid: for example, the thermal conductivity of water/FC72 nanoemulsion containing 12% water nanodroplets by volume with 9.8-nm radius was 52% higher, and approximately 126% increase in effective specific heat was achieved when the water added inside undergo phase change [50, 51]. Pool boiling heat transfer studies of nanoemulsion fluids also show a significant increase in heat transfer coefficient (HTC) and critical heat flux (CHF) compared to base fluid [53, 57, 58].

Convective heat transfer of conventional heat transfer fluids inside micro/minichannel heat exchanger has been intensively studied due to its capability to remove high flux [27, 31, 61–67]. However, relatively few studies have been carried out to investigate the application of novel heat transfer fluids inside mini/microchannels. The recent development of nanotechnology has led to intensify the heat transfer coefficient by using novel nanostructured working fluids. While there are some recent experimental studies, which have demonstrated the possibility of using nanostructured heat transfer fluids inside micro/nanostructured surface to enhance heat transfer [20, 25, 28], other recent studies showed that the use of nanofluids and nanotube coating offers a lower heat transfer coefficient at the coated surface compared to the bare surface [67–70].

Despite the significant enhancement observed in pool boiling heat transfer of nanoemulsion fluids compared to the base fluids, it remains inconclusive whether the same optimistic outlook can be expected in the convective heat transfer of nanoemulsion fluids. In this study, the flow and heat transfer characteristics of ethanol/PAO nanoemulsion fluids under laminar to transitional flow regimes are investigated experimentally.

## Methods

### Nanoemulsion Preparation

**Fig. 1:**
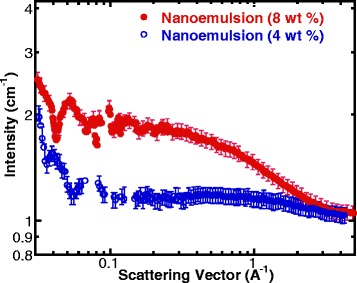
Small-angle neutron scattering curve for 8% (*w*/*w*) and 4% (*w*/*w*) ethanol/PAO nanoemulsion fluids

To minimize the impact of the differences in thermophysical properties of testing fluids on convective heat transfer experiments, ethanol and PAO fluids were used to prepare the nanoemulsion for this study since their thermal conductivity values are very similar. Dioctyl sulfosuccinate sodium salt (Sigma Aldrich) was used as surfactant to form the nanoemulsion. In the preparing process, the first step was dissolving the Dioctyl sulfosuccinate sodium salt into PAO fluid; then, ethanol was injected into the based fluid and mixed well. After that, the mixture became transparent and was thermodynamically stable. In this study, ethanol (8 or 4% of ethanol by weight) is added into PAO to form 8 wt% ethanol/PAO and 4 wt% ethanol/PAO nanoemulsion fluids, respectively. Figure 1 shows the small-angle neutron scattering experimental results of ethanol nanodroplets measured by NG7 small-angle neutron scattering (SANS) instrument at NIST Center for Neutron Research (NCNR), and the data was reduced to extract the structural information following the protocol provided by NCNR [71, 72]. The analysis of the SANS data suggests that the ethanol forms spherical droplets inside the nanoemulsion fluids, which are encapsulated by a layer of surfactant molecules as a core-shell structure [55, 58]. As required by the SANS measurement, the ethanol used for SANS experiment is ethanol-D6 and the measured size of the ethanol nanodroplets (cores) is less than 1 nm in radius on average. The error in droplet size is about 10%. The average size of the ethanol nanodroplets inside the 8 wt% nanoemulsion is larger than those in 4 wt% one (0.8 versus 0.5 nm in radius) as shown in Table 2.

**Table 2:** Thermophysical properties of ethanol/PAO nanoemulsion and PAO at 25 °C

Property	4 wt% nanoemulsion	8 wt% nanoemulsion	PAO
Density (kg/m^3^)	794	792	798
Thermal conductivity (W/m K)	0.148	0.149	0.143
Viscosity (10^−3^ kg/m s)	13.8	14.3	7.34
Nanodroplet radius (nm)	0.5	0.8	N/A

The thermophysical properties of ethanol/PAO nanoemulsion fluid and pure PAO tested in this study are summarized in Table 2.

### Experimental Setup

**Fig. 2:**
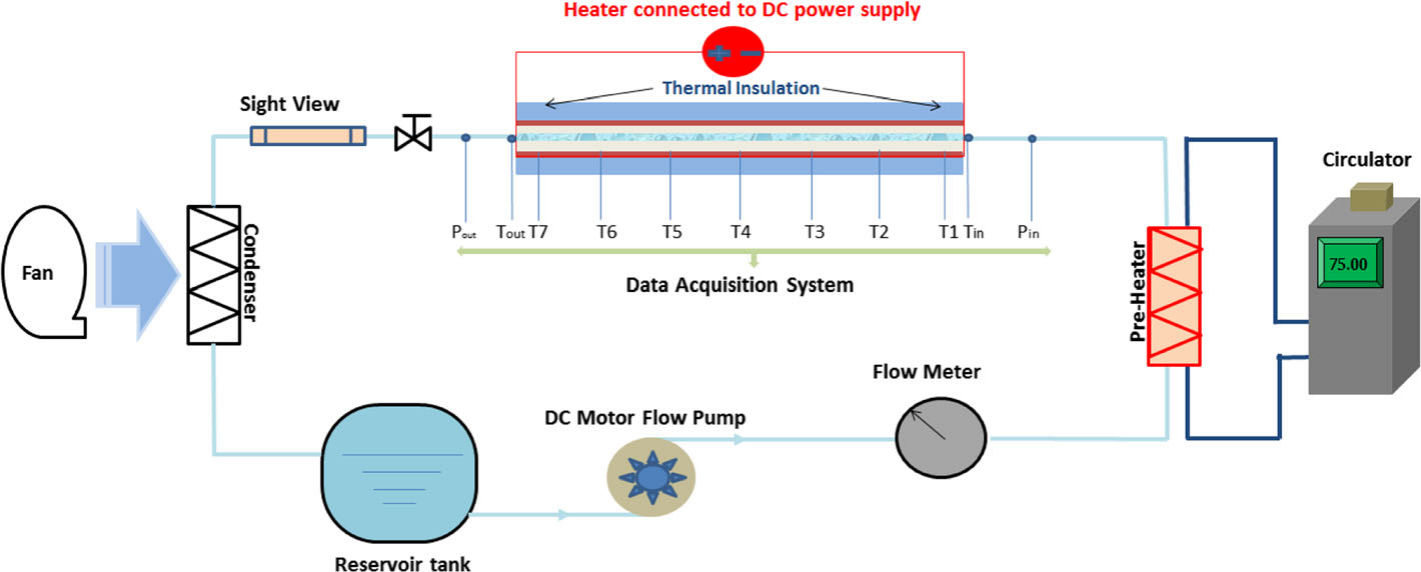
Schematic of the test loop

The convective heat transfer test of nanoemulsion was carried out in a heat exchanger that comprises of 12 minichannels; a schematic diagram of the test loop setup that has been built to conduct experiments is shown in Fig. 2. The test loop is consisted of a horizontal test section of minichannel heat exchanger, three gear pumps, a test fluid tank, a flow sight view, preheating section, condenser, and data acquisition system to measure and record the pressure, temperature, and mass flow rate. In this study, the heat transfer was performed with a constant heat flux applied on the top and bottom surfaces of the minichannel heat exchanger. A programmable DC power supply with 0.05% power uncertainty was used to electrically heat the test section with constant heat flux. The heat flux inputs were varied from 13,000 to 44,000 W/m^2^ to simulate single- and two-phase flow heat transfer test conditions. Moreover, the preheating section was also electrically heated by a circulator, and the inlet fluid temperature is controlled at desired value before entering the minichannels. The inlet and outlet fluid temperatures are measured by two K-type thermocouples. The test section was carefully wrapped with insulation material with a thermal conductivity of 0.043 W/m K. A layer of aluminum foil was then wrapped on the outside of thermal insulation layer. The heat losses through the insulation layer was estimated to be lower than 2% of total heat losses, and it was neglected in the heat transfer coefficient calculation in this study. Pressure drops of the test section were measured by two GP-50 differential pressure transducers with a working range of 0–200 kPa and an uncertainty of 0.25%. For all the tests, the minichannel heat exchanger was placed horizontally to the ground. The liquid in the reservoir was first preheated to a preset temperature of 75 °C. The liquid flow rate was adjusted to the desired value and monitored by a digital paddle wheel flow meter (Micro-Flow™). In the experiment, the fluid temperature and wall temperature are automatically recorded by the data acquisition system. The test system reaches steady state when the changing rates of all the set parameters mentioned above are less than 0.2%. The whole test rig was fully automated using the National Instrument LabVIEW software and data acquisition devices (National Instruments Corp., Austin, TX, USA).

**Fig. 3:**
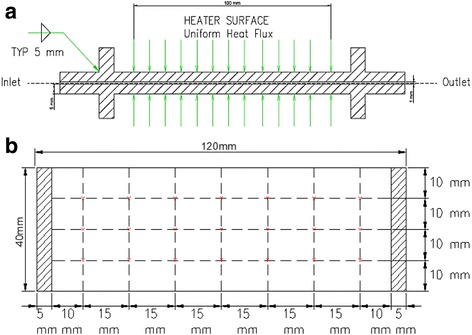
Schematic of the two minichannel heat exchanger. **a** Side view. **b** Top view

Figure 3a, b represents the side view and top view of the minichannel heat exchanger containing 12 minichannels of 1 mm in diameter and 15 cm in length. Twenty-one thermocouples were attached to the top surface of the minichannel heat exchanger and were used to measure the local temperatures as shown in Fig. 3b, in which each red dot represents one thermocouple.

### Data Processing

In this study, the average heat transfer coefficient $h$ is used, and the definition for that is given as follows:

1 $$ h=\frac{q_{\mathrm{wall}}}{T_{\mathrm{m}}-{T}_{\mathrm{f}}} $$

where ${q}_{\mathrm{wall}}$ is the local heat flux estimated by taking into account the local heat loss as shown in Eq. (2), ${T}_m$ is the average local surface temperature measured by the thermocouples along the channel direction calculated by Eq. (3), ${T}_{\mathrm{f}}$ is the fluid bulk mean temperature calculated by Eq. (4).

2 $$ {q}_{\mathrm{wall}}=\frac{Q}{n\left(\uppi Dl\right)} $$

3 $$ {T}_m=\frac{1}{7}{\displaystyle {\sum}_{\mathrm{x}=1}^{\mathrm{x}=7}{T}_{\mathrm{x}}} $$

4 $$ {T}_{\mathrm{f}}=\frac{1}{2}\left({T}_{\mathrm{f},\mathrm{in}}+{T}_{\mathrm{f},\mathrm{out}}\right) $$

in which the $D$ is the inner diameter of the minichannel, $l$ is the length of the minichannel, and $n$ is the number of minichannels.

The average Nusselt number of nanoemulsion flow boiling, based on the inner diameter of the minichannel, can be expressed by

5 $$ \mathrm{Nu}=\frac{hD}{k_{\mathrm{f}}} $$

In this study, flow rate is varied from 0.56 to 14 m/s and Reynolds number of the flow is calculated by

6 $$ \mathrm{R}\mathrm{e}=\frac{\uprho \mathrm{VD}}{\upmu} $$

Total pressure differential is calculated by

7 $$ \Delta P=\Delta {P}_{\mathrm{frictional}}+\Delta {P}_{\mathrm{minor}} $$

and the friction factor is calculated by

8 $$ f=\Delta {P}_{\mathrm{friction}}\frac{2 D}{\rho L{V}^2} $$

### Uncertainty Analysis

The uncertainties for different parameters involved in the experimental test are listed here in Table 3.

**Table 3:** Uncertainties for different parameters involved in the experimental tests

Parameter	Uncertainty
Temperature (°C)	±0.1
Volumetric flow rate, (m^3^/s)	±6%
Position of the thermocouples, (m)	±0.1
Dimensions of the minichannels, (m)	±0.1
Power input, (W)	±0.5%
Heat flux, (W/m^2^)	±5%
Pressure (Pa)	±0.25%

An uncertainty analysis is performed from the measurement uncertainties using calculus and the principle of superposition of errors. In general, for a variable *F* that is a function of several variables such as $F= F\left( a, b, c,\dots \right)$, the squares of the uncertainty in $F$ are the sum of the square of the uncertainties due to each independent variable, $\delta F={\left[{\left(\frac{\partial F}{\partial a}\delta a\right)}^2+{\left(\frac{\partial F}{\partial b}\delta b\right)}^2+{\left(\frac{\partial F}{\partial c}\delta c\right)}^2+\dots \right]}^{0.5}$ where $\delta a$ denotes the uncertainty due to variable *a*. The uncertainties of the calculated variables are shown in Table 4.

**Table 4:** Uncertainties for calculated variables

Calculated variable	Uncertainty
Heat transfer coefficient, *h*(W/(m^2^ K))	±6%
Nusselt Number, $\mathrm{Nu}$	±8%
Reynolds Number, $\mathrm{R}\mathrm{e}$	±6%
Friction factor, $\mathrm{f}$	±10%

## Results and Discussion

### Experimental Results of PAO Fluid

The flow and heat transfer characteristics of the experimental setup were carefully checked to verify the integrity of the experimental facility and test procedures. The test on based PAO fluid was then performed, and the test results were used as baseline data to compare with the test results of ethanol/PAO nanoemulsion fluids. All the experiment performed here was repeated five times, and the relative errors of test data were found to be smaller than 5%. One thing to notice here is that the pure PAO fluid has a boiling temperature above 250 °C, and it remains single phase under all test conditions used in this study.

#### Heat Transfer Experimental Results

**Fig. 4:**
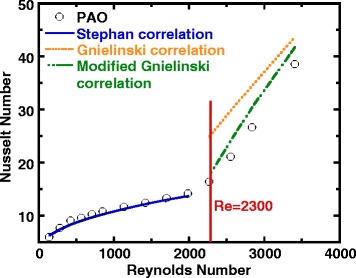
Average Nusselt number versus Reynolds number (pure PAO fluid)

Figure 4 shows the variation of the measured average Nusselt number as a function of Reynolds number for pure PAO, in which the Nusselt number gradually increases when Reynolds number increases. At Reynolds between 2000 and 2200, the Nusselt number starts to increase at a greater rate, which indicates a transition from laminar flow. The results are compared with some classic heat transfer correlations for internal flow as shown in Fig. 4, in which the Stephan correlation is used to describe the internal flow in laminar flow region [73], the Gnielinski correlation was used for fully developed turbulent flow and transitional flow regimes [73], and a modified Gnielinski correlation [74] is used starting from the Reynolds number at the border of laminar to transitional flow regime. It can be seen from Fig. 4 that the experimental results in general agree well with the theoretical predictions in both laminar flow and transition flow regions. However, the modified Gnielinski correlations overestimated the Nusselt number in transtional flow regime. The heat transfer correlations are tabulated in Table 5, together with their corresponding hydrodynamic and thermal conditions and the applicable range.

**Table 5:** Selected conventional heat transfer correlations from the literature

Correlation	Conditions	Range of validity
Stephan correlation [73] $\mathrm{Nu}=4.364+\frac{0.086{\left(\mathrm{Re} \Pr \frac{\mathrm{D}}{\mathrm{L}}\right)}^{1.33}}{1+0.1 \Pr {\left(\mathrm{Re}\frac{\mathrm{D}}{\mathrm{L}}\right)}^{0.83}}$	Laminar flow in a circular pipe	Re < 2300
Gnielinski correlation [73] $\mathrm{Nu}=\frac{\left(\mathrm{f}/8\right)\left(\mathrm{Re}-1000\right) \Pr }{1+12.7\sqrt{\frac{\mathrm{f}}{8}}\left(\mathrm{P}{\mathrm{r}}^{\frac{2}{3}}-1\right)}$ where $f=\frac{1}{{\left(1.82 \log \left(\mathrm{Re}\right)-1.64\right)}^2}$	Constant wall heat flux Fully developed turbulent and transition flow	3000 < Re < 5 × 10^4^
Modified Gnielinski correlation [74] $\mathrm{Nu}=\frac{\left(\mathrm{f}/8\right)\left(\mathrm{Re}-1000\right) \Pr }{1+12.7\sqrt{\frac{\mathrm{f}}{8}}\left(\mathrm{P}{\mathrm{r}}^{\frac{2}{3}}-1\right)}$ where *f* = 3.03 × 10^− 12^ ⋅ Re^3^ − 3.67 × 10^− 8^ ⋅ Re^2^ + 1.46 × 10^− 4^ ⋅ Re − 0.151	Constant wall heat flux Transition flow in straight circular pipe	2300 < Re < 4500

#### Pressure Drop Experimental Results

**Fig. 5:**
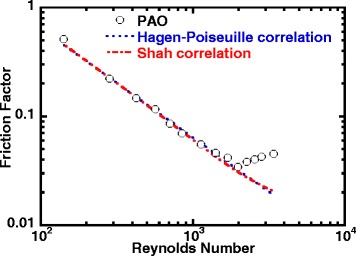
Friction factor versus Reynolds number (pure PAO fluid)

The flow characteristics of pure PAO inside the minichannel heat exchanger are monitored during the experiment, and the friction factor is calculated and shown in Fig. 5 as a function of Reynolds number. The experimental data was compared with the Hagen-Poiseuille correlation for a fully developed laminar flow inside a circular minichannel [73]. Considering the ratio of L/D is relatively large (L/D=150), the Shah correlation was used to include the developing length effect [73]. The friction factor correlations are enumerated in Table 6, together with their corresponding hydrodynamic conditions and the applicable range. As shown in Fig. 5, the friction factor of PAO flowing inside the minichannel heat exchanger agrees well with the classic Hagen-Poiseuille and Shah correlations at laminar flow region and that the frictional factor decreases when Reynolds number increases until Reynolds number reaches around 2000. After that, the friction factor starts to increase sharply when Reynolds number is over 2000, and it keeps increasing until the highest tested Reynolds number. The test results showed both Hagen-Poiseuille and Shah correlations failed to capture the early transition to turbulent flow regime for pure PAO in circular minichannels. The flow characteristics of pure PAO agrees well with the trend observed in the heat transfer data of pure PAO as shown in Figs. 5 and 4, respectively. It is also observed that there is a transition from laminar to turbulent flow when Reynolds number is around 2200.

**Table 6:** Selected friction factor correlations from the literature

Correlation	Conditions	Range of validity
Hagen-Poiseuille correlation [73] *f* ⋅ Re = 64	Laminar flow inside circular channel	Re < 2300
Shah correlation [73] $f\cdot \mathrm{R}\mathrm{e}\approx \left(4\frac{3.44}{\sqrt{\xi}}+\frac{16+0.3125\xi -\frac{3.44}{\sqrt{\xi}}}{1+2.12\times \frac{10^{-4}}{\xi^2}}\right)$ Where $\xi =\left(\frac{\mathrm{x}}{\mathrm{D}}\right)/\mathrm{R}\mathrm{e}$	Laminar flow inside circular channel with consideration of entrance length	Re < 2300

### Experimental Results of Ethanol/PAO Nanoemulsion Fluids

After the integrity of the test loop has been confirmed based on the experimental results of pure PAO fluid, the heat transfer and flow characteristics of ethanol/PAO nanoemulsions with 4 and 8 wt% ethanol were studied following the similar test procedure. A range of heat fluxes from 1.3 to 4.4 kW/m^2^ were selected as heat inputs to simulate single-phase and two-phase convective heat transfer conditions. Each test was repeated at least five times, and the relative differences in measured data are less than 5%. The SANS measurement was performed only to the samples before going through the experiments. However, previous studies on a similar water/PAO system have shown that there are no noticeable structure changes of the nanodroplets before and after pool boiling tests inside an enclosed system [59].

#### Single-Phase Heat Transfer Experimental Results

**Fig. 6:**
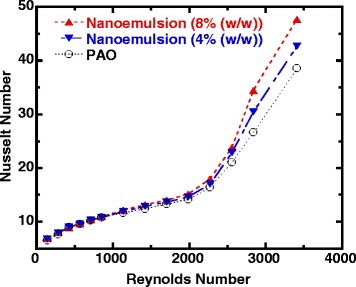
Average Nusselt number versus Reynolds number (nanoemulsion and pure PAO)

Figure 6 provides an overview of the comparison of the measured average Nusselt number for nanoemulsion fluids and the base fluid over the entire range of Reynolds number studied in the present work, in which the variation of the measured average Nusselt number is plotted as a function of Reynolds number. It clearly shows that nanoemulsion fluids can enhance convective heat transfer modestly in laminar flow and a significant heat transfer increase in the transitional and the early stage of turbulent flow. The enhancement in heat transfer in the transitional flow regime can be attributed to the enhanced interaction and interfacial thermal transport between ethanol nanodroplets and base PAO fluid. Previous study has found that the thermal transport between micelles and base fluid is not efficient [75]; it can be enhanced further through better mass exchange and molecular movement at transitional and turbulent flow. This trend has also been demonstrated by increasing nanodroplet concentration at a given Reynolds number: for example, a 24% increase in average Nusselt number for 8 wt% nanoemulsion fluid and a 11% increase in average Nusselt number for 4 wt% nanoemulsion fluid compared to that of pure PAO at the same Reynolds number of 3400. It can be explained by the same hypothesis that the increase in density and size of nanodroplets at higher concentration of ethanol can contribute to a stronger mixing and mass exchange effects at transitional and turbulent flow. It is also worth noting that the Prandtl number (Pr) of ethanol/PAO nanoemulsion (Pr = 42) is higher compared to that of pure PAO (Pr = 31). Furthermore, the nanoemulsion turned from laminar flow to turbulent flow at around the same Reynolds number as for the pure PAO, which agrees well with the fact that the thermophysical properties of nanoemulsion stay very similar to base PAO fluid at room temperature as shown in authors’ previous studies [52, 55], and their difference further decreases when temperature increases and becomes less significant (∆*μ* ≤ 1 × 10^−3^ Pa s at inlet temperature, *T*_in_ = 75 °C).

**Fig. 7:**
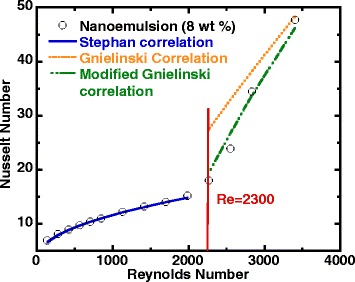
Average Nusselt number versus Reynolds number (nanoemulsion)

Figure 7 shows the comparison of heat transfer data of 8 wt% ethanol/PAO nanoemulsion with conventional heat transfer correlations for internal flow. It can be seen from Fig. 7 that the experimental results agreed very well with the theoretical prediction in both laminar flow and transition flow regions, and the nanoemulsion entered transitional flow region slightly earlier than pure PAO at Reynolds number closer to 2000. It is also shown that while the classic Gnielinski overestimates the Nusselt number for transitional flow, the Stephan and modified Gnielinski correlations provide a better prediction of Nusselt number for transitional flow up to the highest attained Reynolds number (Re = 3400).

#### Single-Phase Pressure Drop Experimental Results

**Fig. 8:**
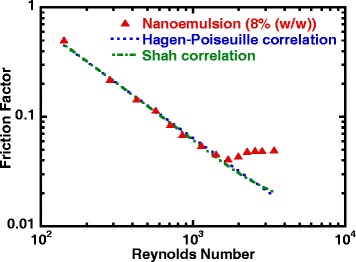
Friction factor versus Reynolds number (nanoemulsion)

Figure 8 shows the measured friction factor for nanoemulsion as a function of Reynolds number, in which it shows very similar trend as observed in the single-phase pure PAO fluid heat transfer data as shown in Fig. 5. Within the laminar flow region, the friction factor of nanoemulsion decreased when Reynolds number increased until Reynolds number just reached 2000, which indicated a slightly earlier entrance into transitional flow compared to that of pure PAO. After the flow entered the transitional flow regime, the frictional factor kept increasing and it started to flat out at Reynolds number close to 3000 which indicates the early entrance into turbulent flow regime. It has also shown a good agreement between the experimental data and the Hagen-Poiseuille and Shah correlations. However, in similar fashion with the case for pure PAO fluid, both correlations failed to show the early entrance into transitional flow for nanoemulsion fluids in circular minichannels.

#### Two-Phase Heat Transfer Experimental Results

One of the reasons of replacing conventional heat transfer fluids with nanoemulsion is under the hypothesis that the heat transfer can be greatly enhanced when the phase changeable nanodroplets undergo nucleation. Previous studies have shown that a significantly improved HTC and CHF can be achieved in nanoemulsion with phase changeable nanodroplets undergo nucleate boiling [53, 57, 58]. In the current study, the ethanol nanodroplets formed inside the nanoemulsion are expected to act as phase change nuclei at elevated temperature during our convective boiling experiments. The maximum flow rate needs to be limited to less than 4.46 m/s (or Re = 1136) to maintain a wall temperature high enough to trigger flow boiling, and all the data shown in this section are collected when the flow remains in laminar flow region. While both nanoemulsion and its base PAO fluid were tested at same Reynolds number and heat input, there is a slight increase in pumping power (~5%) of nanoemulsion since its viscosity is slightly higher compared to pure PAO at current test temperature (75 °C). The overall efficiency of the system using nanoemulsion compared to base PAO fluid is not included in current study.

**Fig. 9:**
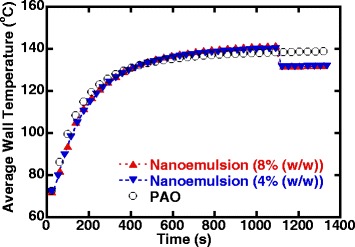
Evolution of the average wall temperature with time (it took about 6000 s for the system to reach steady state, and only stable data is shown in the figure)

Figure 9 shows the transient average wall temperature data for all test fluids (8 and 4 wt%—ethanol/PAO nanoemulsion fluids and pure PAO) with time, which overlapped well with each other in the single-phase flow regime, as shown before in the single-phase heat transfer results. However, the wall temperature of nanoemulsion started to deviate from the single-phase trend line, and a sudden drop in the wall temperature indicates an increase in heat transfer which suggests that it must be induced by factors other than the apparent thermophysical properties, and it was observed that there were bubbles coming out of the minichannel heat exchanger through the sight flow indicator located next to the exit of the minichannel heat exchanger. It confirmed that the ethanol nanodroplets underwent nucleation and the flow inside the minichannel heat exchanger was in flow boiling. While the current setup does not allow an accurate estimation of the amount of ethanol nucleated in the experiment, the amount of ethanol nucleated can be roughly estimated by assuming the additional heat removed is simply caused by the latent heat absorption during the nucleation of ethanol alone: we estimated that approximately 10% of the ethanol inside underwent nucleation during current test conditions. When used in a closed loop, the vaporized ethanol nanodroplets will condense back to liquid phase and form ethanol nanodroplets eventually, because this nanoemulsion system is formed by self-assembly and thermodynamically stable.

Another interesting observation was the delay in nucleation boiling temperature or onset of nucleate boiling (ONB). As shown in Fig. 9, the nucleation did not start until the average surface temperature of the minichannels has reached a temperature close to 140 °C while the boiling temperature for ethanol is 78 °C. Similar findings of delayed ONB were reported in pool boiling heat transfer experiments of sub-cooled ethanol/PAO nanoemulsion fluids [53]. The delayed ONB may also be attributed to the inefficient thermal transport between each surfactant molecule and its surrounding PAO fluid, in which the PAO molecules are not packed closely near the hydrophilic head-group of the surfactant molecule and could not provide efficient thermal pathway in between the micelles and base fluid [76]. Another recent study on similar system also indicates that the nanostructured micelles may experience an endothermic structural change under elevated temperature [77].

**Fig. 10:**
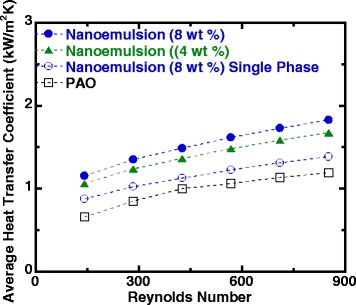
Average heat transfer coefficient versus Reynolds number

The average heat transfer coefficient versus the Reynolds number for flow boiling of nanoemulsion fluids is shown in Fig. 10, in which the single-phase heat transfer data of pure PAO and nanoemulsion are also included for comparison purpose. In addition to the fact that the ethanol/PAO nanoemulsion fluids have slightly higher HTCs compared to pure PAO in single phase due to the minor increase in thermophysical properties, the HTCs can be significantly enhanced when the fluid undergoes connective boiling. It is also apparent that the phase change of the ethanol nanodroplets is the major attribute to the improvement of heat transfer, and the concentration of ethanol inside the nanoemulsion has a positive impact on the overall heat transfer as well. As shown in Fig. 1 of SANS result, the ethanol/PAO nanoemulsion fluid of 8 wt% ethanol has larger droplets inside compared to the 4 wt% one, which may facilitate the packing of PAO molecules at the interface of micelles and PAO and consequently may also provide higher droplets density. This agrees well with our hypothesis that the increase of thermal transport between the nanostructured micelles and base PAO fluid can improve heat transfer coefficient. As shown in Fig. 10, an average increase of 50~70% in HTC can be achieved with ethanol/PAO nanoemulsion compared to that of base PAO fluid.

## Conclusions

In summary, ethanol/PAO nanoemulsion fluid and its convective heat transfer characteristics have been studied experimentally using a heat exchanger made of 12 circular minichannels. Through this special combination of fluids used to form the nanoemulsion, the impact of thermophysical properties difference of the constitutive components on the convective heat transfer of the formed nanoemulsion is minimized, and the experimental results suggest that the interfacial thermal transport and phase change of the ethanol nanodroplets may have a dominating impact on the flow and heat transfer characteristics of nanoemulsion: ethanol/PAO nanoemulsion and PAO exhibit a very similar trend in flow and heat transfer characteristics especially in laminar flow region, which agree well with the fact that they have very similar thermophysical properties. However, the ethanol nanodroplets formed inside the nanoemulsion can be utilized to significantly increase the heat transfer coefficient compared to PAO (e.g., up to 70% increase under flow boiling condition). While the improved heat transfer mechanisms are not completely understood yet, it is hypothesized that the enhancement of heat transfer is likely related to the large latent heat that exists during the phase change of ethanol nanodroplets under flow boiling condition, and also, the enhanced thermal transport between surfactant molecules surrounding ethanol nanodroplets and the base PAO fluid has been shown to be caused by a good mixing and mass transport at transitional and turbulent flow regimes. Overall, this study has shown the possibility of improving flow boiling heat transfer by introducing phase changeable nanostructures into conventional heat transfer fluids, such as the proposed ethanol/PAO nanoemulsion fluids in this study. Studies are urgently needed to further explore the heat transfer enhancement capacity of nanoemulsion fluids, and to obtain a better understanding of its phase change heat transfer characteristics.

## Nomenclature

***Symbols*** **:**

*A* Area, m^2^

*D* Diameter, m

*f* Friction factor

*h* Heat transfer coefficient, W/m^2^ K

*L* Length, m

*n* Number of channels inside the heat exchanger

*Nu* Nusselt number

*P* Pressure, Pa

*Q* Power, W

*q* Heat Flux, W/m^2^

*Re* Reynolds Number

*T* Temperature, K

*V* Velocity, m/s

***Greek Letters*** **:**

*k* Thermal conductivity ratio, W/m K

*ρ* Density, kg/m^3^

μ Dynamic viscosity, Pa s

***Subscripts*** **:**

*f* fluid

*x* location

*in* inlet

*m* mean

*f,in* fluid flow into the minichannels

*f,out* fluid flow out of the minichannels

wall top surface of the minchannels heat exchanger

## Abbreviations

CHF: Critical heat flux
HTC: Heat transfer coefficient
NCNR: NIST Center for Neutron Research
NIST: National Institute of Standards and Technology
Nu: Nusselt number
ONB: Onset of nucleate boiling
PAO: Polyalphaolefin
Pr: Prandtl number
Re: Reynolds number
SANS: Small-angle neutron scattering
